# Supplementary material for: Narrow Precursor Mass Range for DIA–MS Enhances Protein Identification and Quantification in Arabidopsis
Source: Life (Basel). 2021 Sep 18;11(9):982. doi: 10.3390/life11090982 (PMC8469718; doi:10.3390/life11090982)

Supplementary material 1, Example shows how DIA-MS identified and quantified one peptide of ALBU_BOVIN.

1). DIA-MS spectra matching with spectral library and identified a peptide sequence RHPYFYAPELLYYANK (z = 3+) which belongs to the protein ALBU-BOVIN.

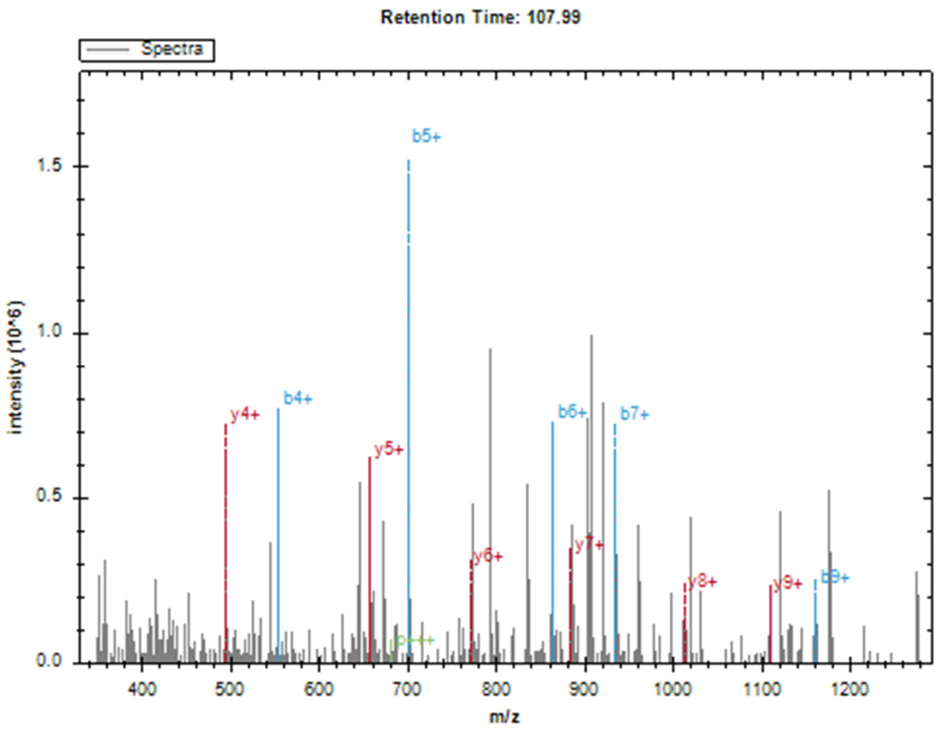

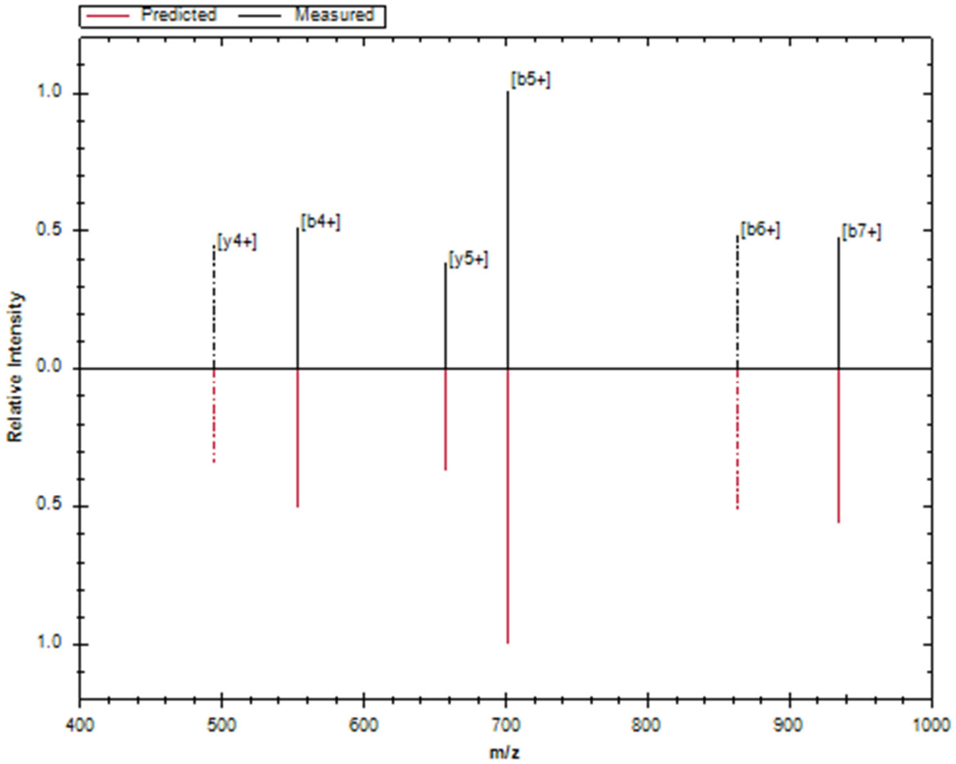

2). MS2 XIC across all runs to generate quantitation information

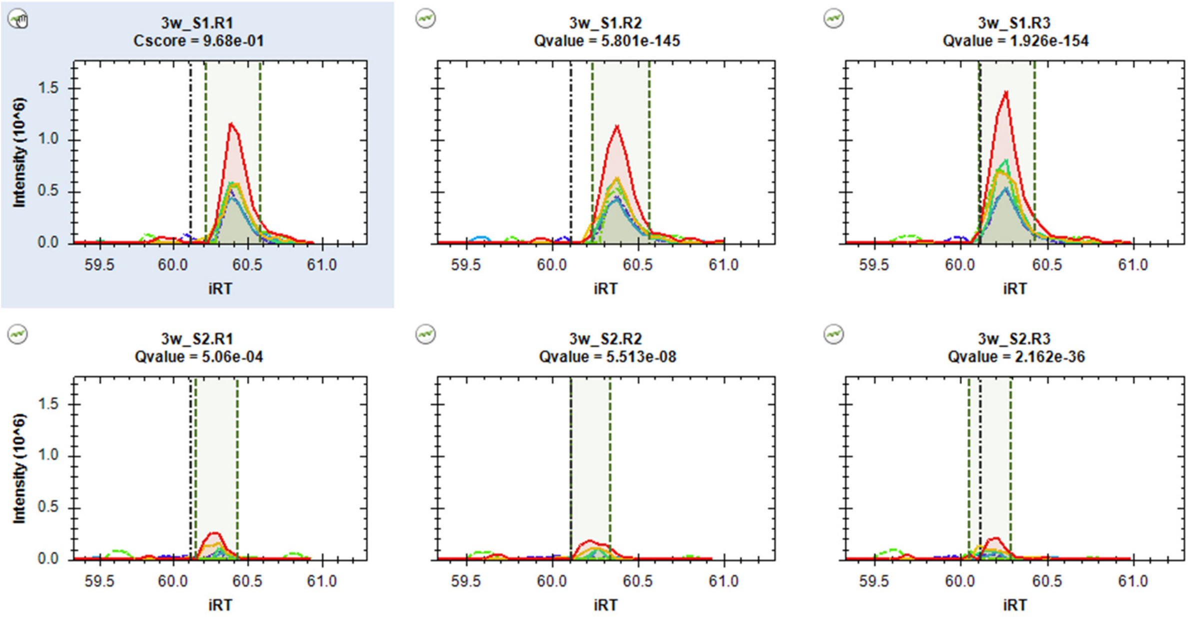

Supplementary material 2. Quantitation of spike-in proteins using cDIA-MS and cGPF-DIA-MS. The upper figure indicates protein quantitation, and the lower panel indicates peptide quantitation for the protein (the dot in the same line represents a same analyte).

<table>
<tr><th>Protein quantitation using cDIA-MS</th><th>Protein quantitation using cGPF-DIA-MS</th></tr>
<tr><td>ALBU_BOVIN</td><td>ALBU_BOVIN</td></tr>
<tr><td>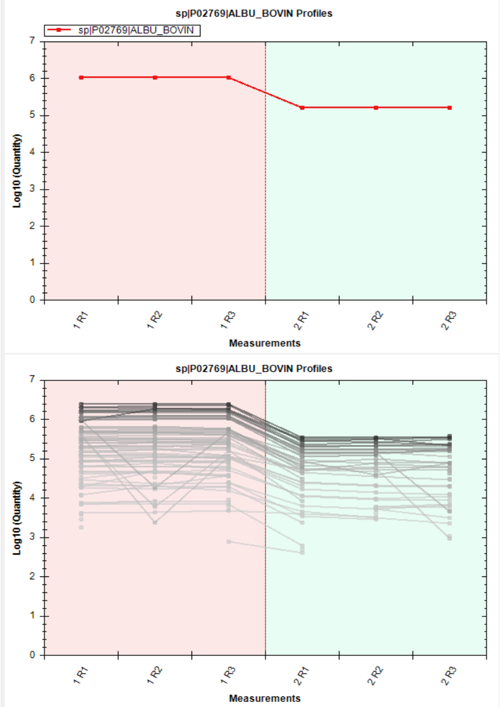
</td><td>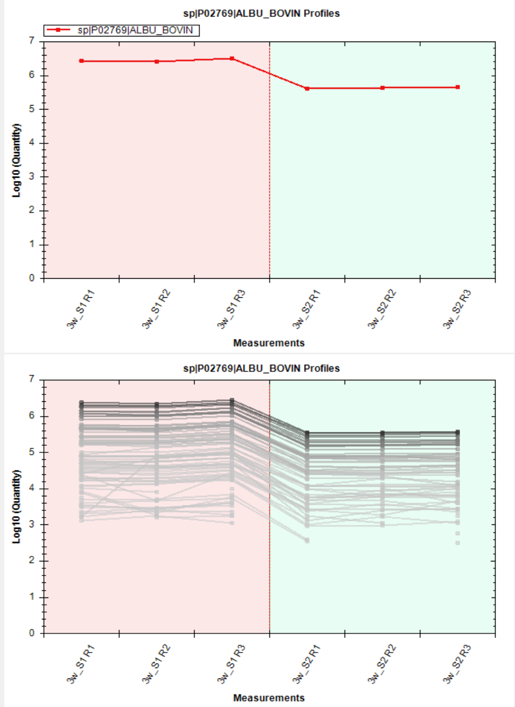
</td></tr>
<tr><td>TRFE_HUMAN</td><td>TRFE_HUMAN</td></tr>
<tr><td>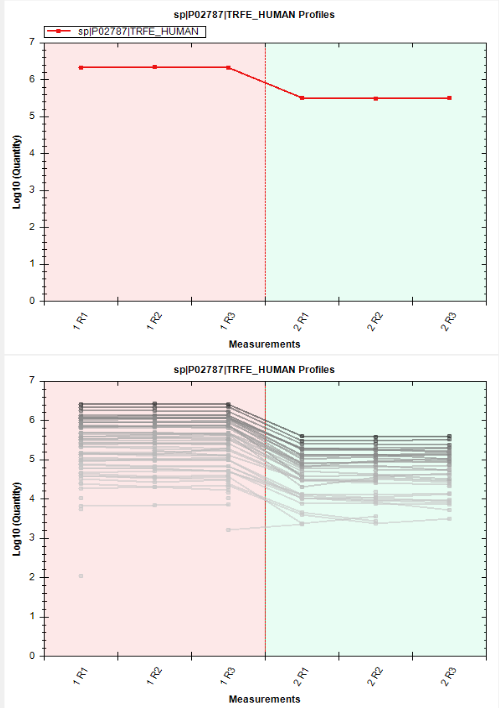
</td><td>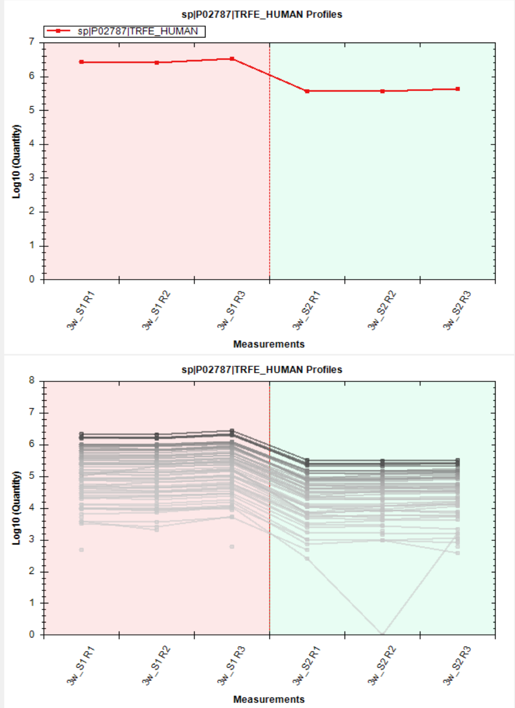
</td></tr>
</table>

OVAL_CHICK	OVAL_CHICK

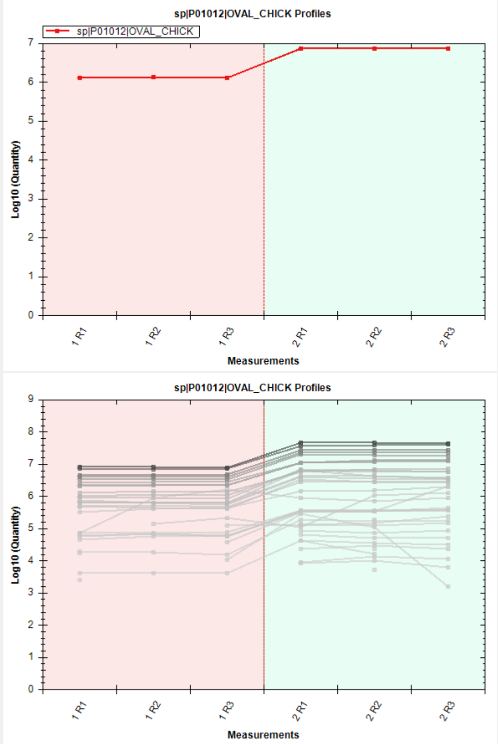

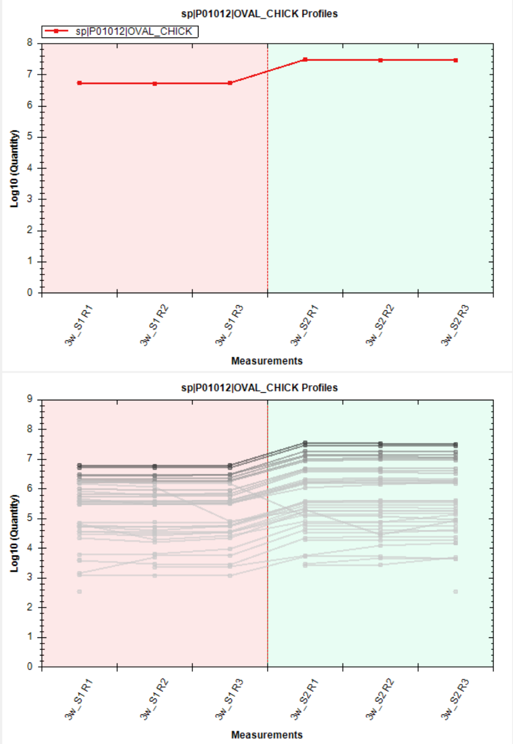

LYSC__CHICK	LYSC__CHICK

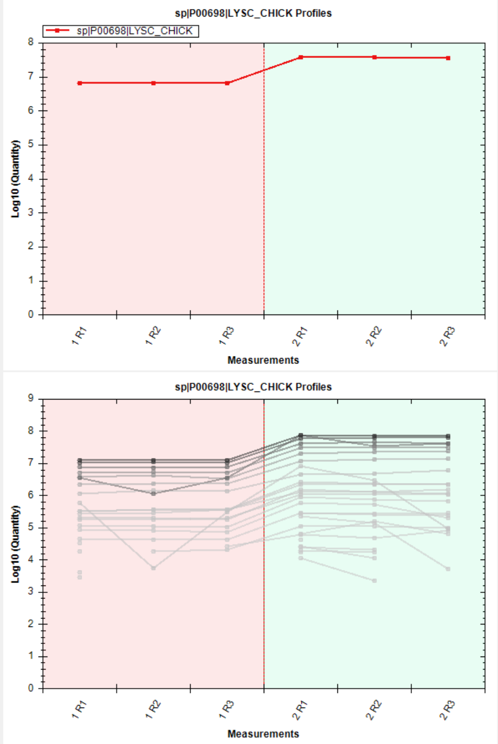

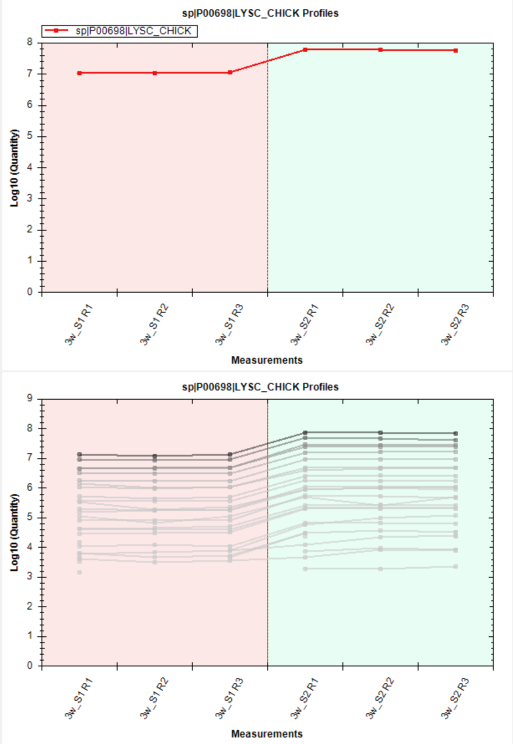

A1AG_BOVIN	A1AG_BOVIN

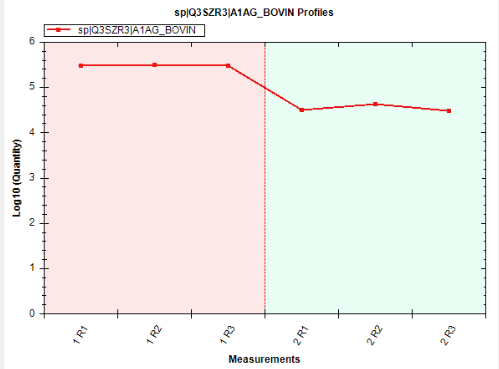 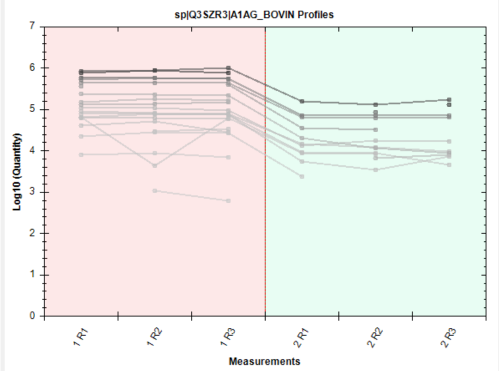	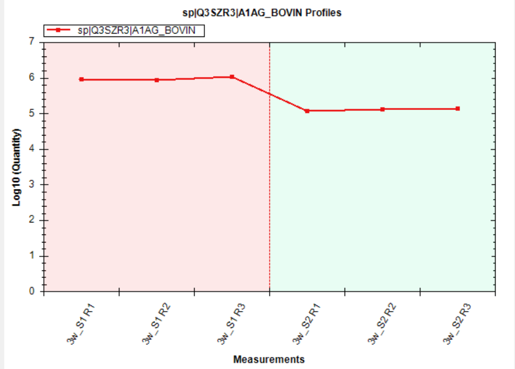 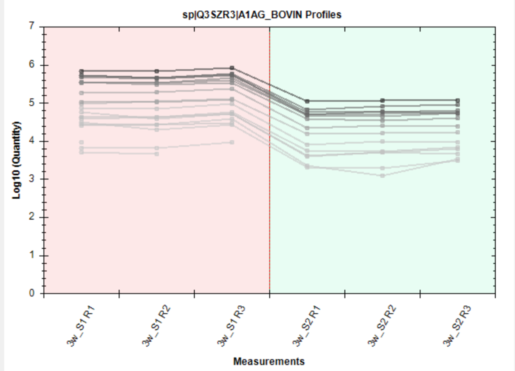
FETUB_BOVIN	FETUB_BOVIN
Not detected as significant change	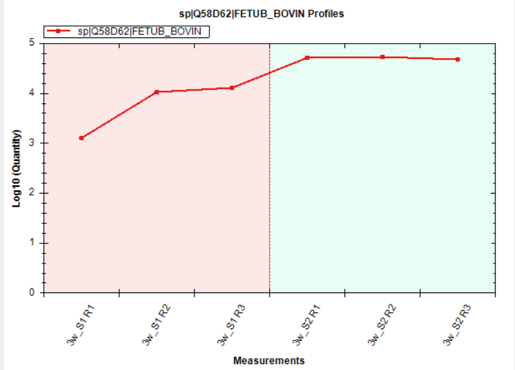 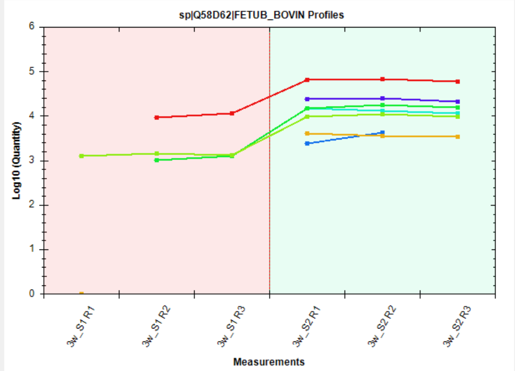

Supplementary material 3, venn diagram shows the protein identified from cGPF-DIA-MS and cDIA-MS analysis.

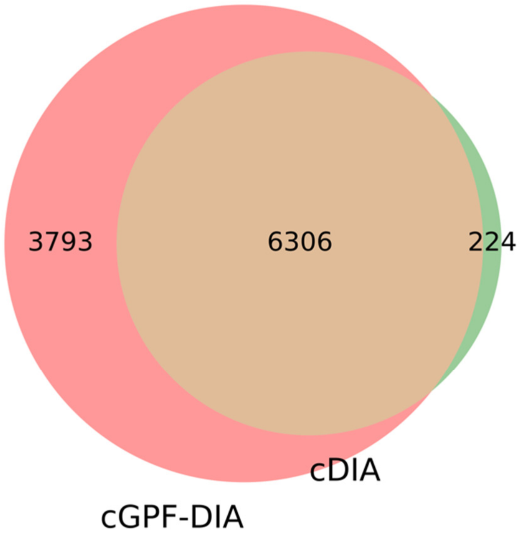

Supplement: Supplementary file 1 [file life-11-00982-s001.zip › Supplemental_materials/Supplementary material.pdf]
